# A Bovine Enteric *Mycobacterium* Infection Model to Analyze Parenteral Vaccine-Induced Mucosal Immunity and Accelerate Vaccine Discovery

**DOI:** 10.3389/fimmu.2020.586659

**Published:** 2020-11-23

**Authors:** Antonio Facciuolo, Amy H. Lee, Michael J. Trimble, Neil Rawlyk, Hugh G. G. Townsend, Manjeet Bains, Natasa Arsic, Lucy M. Mutharia, Andrew Potter, Volker Gerdts, Scott Napper, Robert E. W. Hancock, Philip J. Griebel

**Affiliations:** ^1^ Vaccine and Infectious Disease Organization—International Vaccine Centre (VIDO-InterVac), University of Saskatchewan, Saskatoon, SK, Canada; ^2^ Department of Molecular Biology and Biochemistry, Simon Fraser University, Burnaby, BC, Canada; ^3^ Centre for Microbial Diseases and Immunity Research, Department of Microbiology and Immunology, University of British Columbia, Vancouver, BC, Canada; ^4^ Department of Molecular and Cellular Biology, University of Guelph, Guelph, ON, Canada; ^5^ Department of Biochemistry, Microbiology, and Immunology, University of Saskatchewan, Saskatoon, SK, Canada; ^6^ School of Public Health, University of Saskatchewan, Saskatoon, SK, Canada

**Keywords:** *Mycobacterium*, paratuberculosis, Peyer’s patches, innate lymphoid cell, vaccine, mucosal immunity, interleukin-21, interleukin-27

## Abstract

Mycobacterial diseases of cattle are responsible for considerable production losses worldwide. In addition to their importance in animals, these infections offer a nuanced approach to understanding persistent mycobacterial infection in native host species. *Mycobacterium* *avium* ssp. *paratuberculosis* (MAP) is an enteric pathogen that establishes a persistent, asymptomatic infection in the small intestine. Difficulty in reproducing infection in surrogate animal models and limited understanding of mucosal immune responses that control enteric infection in the natural host have been major barriers to MAP vaccine development. We previously developed a reproducible challenge model to establish a consistent MAP infection using surgically isolated intestinal segments prepared in neonatal calves. In the current study, we evaluated whether intestinal segments could be used to screen parenteral vaccines that alter mucosal immune responses to MAP infection. Using Silirum^®^ – a commercial MAP bacterin – we demonstrate that intestinal segments provide a platform for assessing vaccine efficacy within a relatively rapid period of 28 days post-infection. Significant differences between vaccinates and non-vaccinates could be detected using quantitative metrics including bacterial burden in intestinal tissue, MAP shedding into the intestinal lumen, and vaccine-induced mucosal immune responses. Comparing vaccine-induced responses in mucosal leukocytes isolated from the site of enteric infection versus blood leukocytes revealed substantial inconsistences between these immune compartments. Moreover, parenteral vaccination with Silirum did not induce equal levels of protection throughout the small intestine. Significant control of MAP infection was observed in the continuous but not the discrete Peyer’s patches. Analysis of these regional mucosal immune responses revealed novel correlates of immune protection associated with reduced infection that included an increased frequency of CD335^+^ innate lymphoid cells, and increased expression of *IL21* and *IL27*. Thus, intestinal segments provide a novel model to accelerate vaccine screening and discovery by testing vaccines directly in the natural host and provides a unique opportunity to interrogate mucosal immune responses to mycobacterial infections.

## Introduction

The majority of pathogenic mycobacteria are mucosal pathogens that establish a chronic state of infection. *Mycobacterium* *avium* ssp. *paratuberculosis* (MAP) – the etiological agent of Johne’s disease in ruminants – is an intracellular pathogen that establishes a persistent infection in the small intestine. With no effective vaccine to control or prevent infection, Johne’s disease remains endemic in cattle, sheep and goats (1) and contributes to significant global production losses (2–4). Prolonged transmission (5) of MAP in feces (6) and milk (7) by asymptomatic carriers impedes current efforts to reduce MAP prevalence in dairy and beef cattle. Moreover, detection of MAP in the environment, retail milk, and water supplies coupled with associations with Crohn’s disease in humans have raised food safety concerns of MAP zoonosis (8). The development of an effective MAP vaccine remains an elusive goal despite decades of research (9) primarily due to the lack of understanding the mucosal immune responses that control enteric MAP infections within the natural host (i.e., cattle, goat, and sheep). Such analyses may also provide new insights into mucosal mycobacterial diseases in humans and other animal host species (10).

Three vaccines have been licensed for the control of Johne’s disease. Mycopar^®^ (Boehringer Ingelheim, USA) was licensed in the United States for use in cattle. Silirum^®^ (CZ Veterinaria, S.A., Spain) is licensed in Spain for use in sheep, and Gudair^®^ (Zoetis, USA) is licensed in Australia for use in sheep. These vaccines are all inactivated bacterins formulated in oil emulsions and delivered by parenteral injection. Most countries and producers are hesitant to implement these vaccines since there is the potential for MAP bacterins to induce cell-mediated and antibody responses that cross-react with antigens used for bovine tuberculosis testing (11–13). Furthermore, these vaccines do not prevent or control infection but have vaccine claims for a reduction in clinical disease and fecal shedding of MAP (9, 14–16). Experimental alternatives to the inactivated bacterins have been developed and evaluated. These include parenteral (17) and oral (11) live-attenuated vaccines, parenteral subunit vaccines containing recombinant MAP proteins (18, 19), and parenteral viral vectored vaccines (20). These experimental vaccines have been evaluated in either caprine (17, 21) or bovine (19, 20) oral MAP challenge models. Parenterally administered vaccines can alter the host-MAP interaction at a mucosal site but fail to achieve substantial reductions in either intestinal infection or MAP fecal shedding, parameters essential for effective control of Johne’s disease.

Failure to develop an effective vaccine to control MAP infection has not been due to a lack of candidate MAP antigens. Advances in bioinformatics and recombinant technologies has provided a growing list of novel MAP vaccine antigens and live-attenuated strains. Effective screening of vaccine candidates has been limited by a lack of validated cellular and cytokine immune correlates of protection. This has in turn limited rapid screening of potential vaccine candidates. Moreover, animal models for vaccine screening, such as mice and goats, fail to recapitulate bovine MAP enteric infection. For example, in the goat model, persistent MAP infection is consistently established in major enteric mucosal immune induction sites such as discrete Peyer’s patches (DPP) in the jejunum, the ileocecal valve PP and the proximal colon PP but is not consistently present in the continuous PP (CPP) (22, 23). In contrast, MAP is consistently detected in CPP in both naturally infected (24, 25) and experimentally challenged calves and cows. Furthermore, when comparing calf and goat oral infection models, significant inter-species differences in MAP bacterial burden in intestinal tissues and mesenteric lymph nodes were reported (26).

We developed a surgical model in neonatal calves to establish consistent levels of MAP infection at specific intestinal sites (27–29). With this infection model we observed MAP infection persists in CPP but is significantly reduced or controlled in DPP by 12 months post-infection (29). Consistent infection in our model overcomes the problem of variable infection rates and non-uniform distribution of infection throughout the gastrointestinal tract observed with oral challenge models (11, 19–21, 30–32). Further, oral challenge is associated with intermittent fecal shedding occurring days, weeks, and months post-infection (33, 34). Fecal shedding may result in recurrent enteric infection at multiple sites throughout the gastro-intestinal tract. These factors increase inter-animal variability following oral challenge and impedes quantification of MAP intestinal burden, fecal shedding, and mucosal immune responses within and among experimental groups. Furthermore, oral infection models are expensive, have a prolonged infection period to measurable clinical disease, and in many cases do not use the natural target host species. Challenges associated with sampling intestinal tissues and isolating mucosal leukocytes from a known site of infection to analyze local immune responses present additional hurdles that have hindered characterization of vaccine antigen immunogenicity and efficacy. The majority of MAP vaccine studies have used blood leukocytes to analyze immune responses with no validation that similar immune responses occur at the site of enteric infection. In the absence of data linking systemic and mucosal immune responses, there is no rational basis for evaluating parenterally delivered vaccine antigens or vaccine formulations.

There is a need for a model in the natural host that achieves consistent levels of infection in all challenged animals, provides quantitative metrics of vaccine efficacy, and is amendable to high throughput analysis to accelerate screening of candidate MAP vaccines. In this study, we used a surgical infection model in neonatal calves as a model for screening parenteral vaccines, and investigated whether vaccination controlled enteric infection and altered mucosal immune responses following enteric MAP infection. Silirum^®^, a MAP bacterin that is injected parenterally, demonstrated proof-of-concept that intestinal segments provide a platform that could be used to assess vaccine efficacy within 28 days post-infection. Quantitative metrics including bacterial burden in intestinal tissue, MAP shedding into the intestinal lumen and vaccine-induced mucosal immune responses were used to determine if significant differences could be detected when comparing vaccinated and unvaccinated controls. We demonstrate with both Silirum and recombinant MAP protein-based vaccines that vaccine-induced responses occurring at the site of enteric infection did not reflect responses measured with blood leukocytes. Furthermore, we observed that Silirum vaccination did not induce equal levels of protection throughout the small intestine. Regional differences in control of MAP infection provided an opportunity to identify mucosal immune responses correlating with control of enteric MAP infection in the natural host. We utilized flow cytometric analysis and analysis of vaccine-induced cytokine responses to identify novel surrogate markers of immune protection. The potential for this novel MAP infection model to accelerate MAP vaccine discovery and further our understanding of immune responses controlling mycobacterial mucosal infections are discussed.

## Materials and Methods

### Ethics Approval Statement

All animal experiments were completed at the University of Saskatchewan following guidelines provided by the Canadian Council on Animal Care and approved by the University of Saskatchewan Animal Care Committee (Protocol #20160076).

### Vaccine Study Design

**Figure 1:**
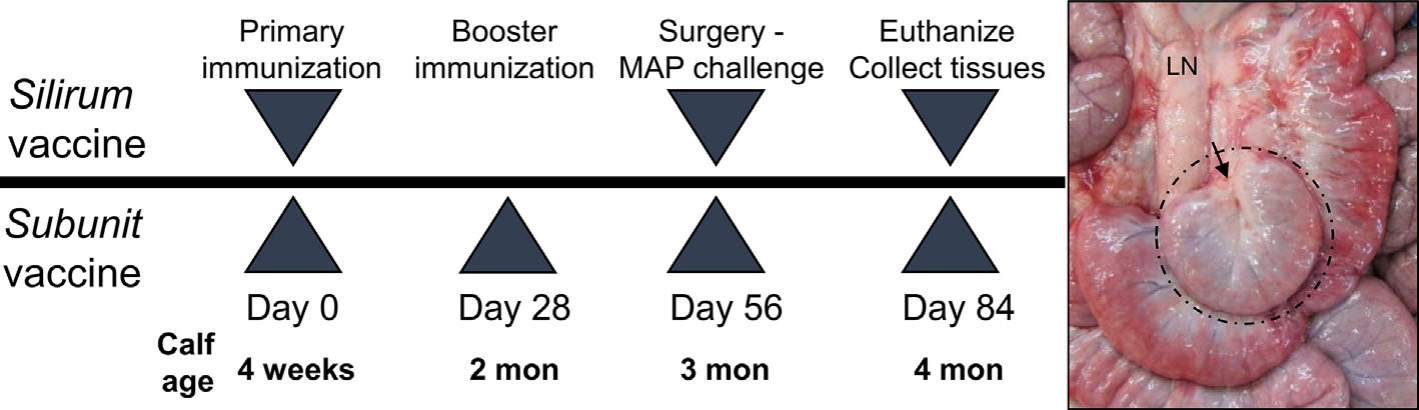
Vaccine study designs. Silirum vaccine study: Six, 4-week old calves received a single subcutaneous dose of the Silirum^®^ vaccine (CZ Veterinaria S.A., Spain) and six other calves received adjuvant alone (MONTANIDE™ ISA 61 VG). Subunit vaccine study: Six calves were subcutaneously injected with adjuvant alone (30% Emulsigen™, 250 µg CpG ODN) and an additional three groups (six calves per group) were each injected with one of three pools of 5 recombinant *Mycobacterium* *avium* ssp. *paratuberculosis* (MAP) proteins using a homologous prime-boost regimen. On Day 56 intestinal segments were surgically prepared and 10^10^ CFU MAP injected into the lumen. A representative *in situ* image of an intestinal segment at 28 days post-infection (Day 84) in a four month old calf is shown with hash marks identifying the intestinal segment located among the surrounding intact intestine and the arrow indicating the site of surgical anastomosis. LN, mesenteric lymph node.

For the Silirum**^®^** vaccine study, twelve (12) male Holstein calves (one month old) sourced from a local supplier were randomly assigned to one of two groups (**Figure 1**). On Day 0, Group 1 (n=6) received a 1 ml subcutaneous injection of the Silirum**^®^** vaccine (CZ Veterinaria, S.A., Spain) in the neck as per the manufacturer**’**s instructions, and Group 2 (n=6) received a sham vaccine prepared with 0.5 ml adjuvant (MONTANIDE™ ISA 61 VG; Seppic S.A., France) mixed with 0.5 ml phosphate-buffered saline (PBS). On Day 56 post-vaccination, intestinal segments were surgically prepared in each calf and challenged with MAP (see *Animals, Surgery and MAP Infection*). On Day 84 (28 days post-infection), blood was collected to isolate peripheral blood mononuclear cells (PBMC), and calves were euthanized to collect intestinal tissue and isolate mucosal leukocytes (see *Isolation of Immune Cells*).

For the recombinant MAP protein-based subunit vaccine study, twenty-four (24) male Holstein calves (one month old) sourced from a local supplier were randomly assigned to one of four groups (**Figure 1**). Group A (n=6) calves were vaccinated subcutaneously with two ml adjuvant (30% Emulsigen; Phibro Animal Health, Omaha, NE) supplemented with 125 µg/ml CpG oligodeoxynucleotides ODN_2007 (BioSpring, GmbH, Frankfurt, Germany). Group B (n=6), group C (n=6) and group D (n=6) calves were vaccinated subcutaneously with a unique pool (50 µg each) of five recombinant MAP proteins (**Table 1**) formulated in 30% Emulsigen and CpG ODN. This vaccine adjuvant formulation has been shown to enhance the immunogenicity of recombinant proteins and promote cell-mediated immune responses when administered parenterally in calves (35). A homologous prime-boost regimen was used with each calf receiving a primary vaccination on Day 0 and a booster on Day 28. On Day 56 post-vaccination, intestinal segments were surgically prepared in each calf and challenged with MAP (see *Animals, Surgery and MAP Infection*). On Day 84 (28 days post-infection), blood was collected to isolate PBMCs, and calves euthanized to collect intestinal tissue and isolate mucosal leukocytes (see *Isolation of Immune Cells*).

**Table 1:** Vaccine-induced cytokine responses in peripheral blood and mucosal leukocytes at 28 days post-infection.

Vaccine Group	MAP load in luminal contents^a,b^	Protein	PBMCs		Discrete PP cells		
			*IFNG* ^c^	*IL17A* ^c^	*IFNG* ^c^	*IL17A* ^c^	*IL12B* ^c^
**B**	**5.75 ± 0.62**	**MAP0367**		5.7 ↑ (p=0.024)			
		**MAP3836c**		5.5 ↑ (p=0.022)			
		**MAP1981c**		7.3 ↑ (p=0.015)		7.0 ↓ (p=0.006)	
		**MAP4143**	5.8 ↑ (p=0.044)	5.9 ↑ (p=0.013)	1.8 ↑ (p=0.0005)		
		**MAP2121c**			1.7 ↑ (p=0.0001)		
**C**	**6.53 ± 0.46**	**MAP1588c**		5.1 ↑ (p=0.020)	1.9 ↑ (p=0.034)		
		**MAP3840**			1.6 ↑ (p=0.062)		
		**MAP4142**	4.1 ↑ (p=0.08)	6.8 ↑ (p=0.013)			
		**MAP1530**					
		**MAP3968**		4.2 ↑ (p=0.030)	1.9 ↓ (p=0.074)		
**D**	**5.37 ± 0.86**	**MAP0144**		2.9 ↑ (p=0.026)	2.1 ↓ (p=0.035)	15.8 ↓ (p=0.0002)	1.3 ↓ (p=0.028)
		**MAP4263**		3.1 ↑ (p=0.049)	2.1 ↓ (p=0.019)	15.2 ↓ (p=0.0006)	1.6 ↓ (p=0.001)
		**MAP3550**		2.7 ↑ (p=0.069)			1.8 ↓ (p=0.003)
		**MAP0115**		3.2 ↑ (p=0.052)	2.4 ↓ (p=0.006)	14.8 ↓ (p=0.003)	2.1 ↓ (p=0.008)
		**MAP0187c**			1.7 ↓ (p=0.037)	8.8 ↓ (p=0.027)	1.6 ↓ (p=0.061)
**A**	**5.73 ± 1.0**						

PBMCs (5 x 10^6^) and Discrete PP cells (2 x 10^6^) collected at 28 days post-MAP infection from non-vaccinates (Group A) and vaccinates (Groups B, C, and D) were re-stimulated ex vivo with individual recombinant MAP protein (2.5 µg/ml) for 24 h. Cytokine transcript abundances of IFNG, IL12B, IL17A, and IL27 were quantified using real-time qRT-PCR.

^a^Mean MAP CFU (log10)/g ± SD.

^b^One-way ANOVA, p=0.22. Group A, n=6; Group B, n=5; Group C, n=4; and Group D, n=6.

^c^Mean fold-difference in transcript abundance of vaccinates relative to non-vaccinates with arrows indicating vaccine-induced upregulation or downregulation. Differences in vaccine-induced cytokine responses relative to non-vaccinates were determined using a Student’s t test.

### Production of Recombinant MAP Proteins

MAP genes, codon optimized for protein expression in *E. coli*, were synthesized as double-stranded DNA fragments (GeneArt Gene Synthesis; Thermo Fisher Scientific, Inc.) for direct cloning into the Gateway^®^ expression vector pET301/CT-DEST (Thermo Fisher Scientific, Inc.). Recombinant plasmids were transformed into *E.coli* BL21Star (DE3) competent cells (ThermoFisher Scientific, Inc.) and verified by sequencing. *E. coli* cells containing recombinant plasmids were cultured in Lysogeny Broth supplemented with 100 µg/ml carbenicillin (MilliporeSigma Canada Co.) at 37°C to an OD_600_ of 0.5-0.6. Recombinant protein expression was induced by adding 1 mM isopropyl β-d-1-thiogalactopyranoside (IPTG; Life Technologies), and bacterial cultures incubated for 4 h at 37°C. Bacterial cells were harvested by centrifugation and the cell pellets suspended in lysis buffer (8 M urea, 500 mM NaCl, 100 mM NaH_2_PO_4_, 10 mM imidazole, and 10 mM Tris-HCl, pH 8) and homogenized by sonication. The homogenate was centrifuged for 10 min at 10,000 x g and the clarified supernatant incubated with nickel-NTA agarose resin (Qiagen, Inc.) for 16–24 h at 4°C to capture C-terminal fused 6xHis-tagged recombinant protein. The resin was packed into a Poly-Prep chromatography column (Bio-Rad Laboratories, Inc.) and washed with four bed volumes of lysis buffer followed by eight bed volumes of wash buffer (8 M urea, 500 mM NaCl, 100 mM NaH_2_PO_4_ and 10 mM Tris-HCl, pH 6.3). Recombinant proteins were eluted from the resin by sequentially adding 1 bed volume of each buffer: Buffer D (8 M urea, 500 mM NaCl, 100 mM NaH_2_PO_4_, 8% glycerol and 10 mM Tris-HCl, pH 5.5); Buffer E (Buffer D, pH 4.5); and 10 mM Tris-HCl, pH 8.0 containing 25 mM EDTA. Protein integrity and purity in each fraction was assessed by SDS-PAGE, and quantified using the Bio-Rad Protein Assay Kit™ (Bio-Rad Laboratories, Inc.). Elution fractions were stored at −80°C.

### Animals, Surgery, and MAP Infection

Conventionally reared, 14-21 day old male Holstein calves were obtained from a local supplier that sources bull calves at 1 day of age from multiple dairy herds in Saskatchewan. Calves were individually housed at the local supplier’s farm. Calves arriving at the facility were acclimated for a minimum of one week. Calves received colostrum from their dams at the time of birth, and were fed commercial milk replacer until weaned at 8 weeks of age. After weaning, calves were fed a diet of hay and a grain-pellet mixture *ad libitum*. Currently, no regional Johne’s disease control programs are active in Saskatchewan, therefore MAP-infection status of dams and colostrum are unknown. Calves were individually housed up to the time of surgery, and then group housed following MAP challenge. Targeted delivery of MAP inoculum into surgically isolated intestinal segments precludes MAP fecal shedding and mitigates any concern of cross-contamination among calves.

Calves were recruited as pairs and randomly assigned to groups. Surgeries were performed on two calves per day over three consecutive days resulting in a weekly cohort consisting of unvaccinated and vaccinated animals. Surgeries were completed weekly until all animals were challenged. Each intestinal segment was approximately 15-20 cm in length. Animal housing, feeding, anesthesia, surgery, post-surgical care, and MAP infection have been previously described in detail (28, 36). Special precaution was taken during each surgery to preserve vasculature and lymphatic connections of each intestinal segment through the mesenteric attachment and continuity of the intestinal tract was re-established by an end-to-end anastomosis of the intestine proximal and distal to the intestinal segment. Calves in the Silirum vaccine study had two intestinal segments surgically isolated: one in the mid-jejunum containing a DPP and the other proximal to the ileocecal fold (terminal jejunum) containing a CPP. Calves in the subunit vaccine study had a single intestinal segment in the mid-jejunum (containing a DPP) surgically prepared. MAP strain gc86 (10^10^ CFU; **Supplementary Figure 1**) was injected into the lumen of each intestinal segment. This strain is a low-passage Type II field isolate of MAP (37) previously used in calf infection studies (28, 29, 38, 39). For each trial, a low-passage frozen stock of MAP strain gc86 was plated and a single colony inoculated into Difco™ Middlebrook 7H9 broth (BD and Company, USA) supplemented with 10% BBL™ Middlebrook OADC Enrichment (BD and Company, USA) and 2 mg/L ferric mycobactin J (Allied Monitor Inc., USA). Log phase cultures were used to prepare cell pellets weighing 300 mg, and aliquots cryopreserved in 7H9 broth containing 25% glycerol and stored at -80°C. One day prior to challenge, an aliquot was suspended in 5 ml of 7H9 medium and incubated at 37°C. At the time of challenge, the MAP cells were re-suspended in 5 ml PBS and used as the inoculum; a sample was serially diluted and plated on Difco™ Mycobacteria 7H11 Agar (BD and Company, USA) supplemented with 10% BBL™ Middlebrook OADC Enrichment (BD and Company, USA) and 2 mg/L ferric mycobactin J (Allied Monitor Inc., USA) to enumerate viable CFUs for each challenge inoculum (**Supplementary Figure 1**).

### Gross Examination and Histology

At 28 days post-infection, calves were euthanized by intravenous injection with Euthanyl (20 ml/45 kg body weight; Bimeda-MTC, Canada) and tissue was collected within 10-15 min. Adjacent intact intestine and intestinal segments were harvested and photographed to document gross appearance. Intestinal segments were bisected along the mesenteric attachment to reveal the mucosal surface, which was examined and photographed, and luminal contents collected. Intestinal tissues were immediately submerged in 10% neutral-buffered formalin for histology. Tissue embedding, tissue sectioning, hematoxylin & eosin (H&E) staining, Fite’s stain (for *in situ* detection of acid-fast bacilli), and immunohistochemical staining (for MAP antigen) was completed by Prairie Diagnostic Services (Saskatoon, SK, Canada). Images of stained tissue sections were acquired using an Olympus Virtual Slide Scanning Microscope (Olympus-Life Science, Japan) which was completed by the WCVM Imaging Centre (University of Saskatchewan, Saskatoon, SK, Canada).

### MAP Bacterial Culture

PP tissue (minimum 100 mg) collected from each intestinal segment was weighed and washed with three exchanges of PBS, vigorously vortexing each time for 20 s to clean the mucosal surface. Tissue was subsequently homogenized in 5 ml PBS using a PRO200 homogenizer (PRO Scientific Inc., USA). Tissue homogenate was serially diluted 10-fold and the first four dilutions were plated on Difco™ Mycobacteria 7H11 Agar (BD and Company, USA) supplemented with 10% BBL™ Middlebrook OADC Enrichment (BD and Company, USA), 2 mg/L ferric mycobactin J (Allied Monitor Inc., USA), 50 µg/ml carbenicillin (MilliporeSigma Co.) and 10 µg/ml amphotericin B (MilliporeSigma Co.). MAP recovery from tissue was calculated as CFU/g of PP tissue. To determine MAP load in the intestinal lumen, luminal contents (approximately 5 g) were weighed, suspended in 30 ml PBS and vigorously vortexed to obtain a homogeneous suspension. This suspension was serially diluted 10-fold and the first 4 dilutions plated. MAP recovery from luminal contents was calculated as CFU/g of contents. All plates were incubated at 37°C, and monitored weekly for 8 weeks to allow for colony formation.

### Isolation of Immune Cells

Blood was collected from the jugular vein and PBMCs isolated using previously established protocols (27). PBMCs were re-suspended in HyClone™ Dulbecco’s Low Glucose Modified Eagles medium (DMEM; GE Healthcare Life Science) supplemented with 10% fetal bovine serum (FBS), 100 µg/ml streptomycin, 100 units/ml penicillin, 0.25 µg/ml amphotericin B (MilliporeSigma Co.), and 10 µg/ml gentamicin (MilliporeSigma Co.; ‘complete DMEM’). Mucosal leukocytes were isolated from the submucosa of PPs following a previously established protocol (28) that recovers cells from the submucosal lymphoid follicles and interfollicular regions of PPs, collectively referred to as ‘PP cells’. The resulting cell suspensions were adjusted to 2.5 x 10^7^/ml in complete DMEM.

Viability of PBMCs and PP cells were consistently > 98% as determined by trypan blue exclusion. For flow cytometric analysis, cells were cryopreserved in aliquots of 2.5 x 10^7^ cells/ml/vial in DMEM medium containing 10% v/v DMSO and 20% FBS.

### *Ex Vivo* Restimulation Assay

Freshly isolated PBMCs (5 x 10^6^/well) and PP cells (2 x 10^6^/well) were seeded in 12-well tissue culture plates in a final volume of 1 ml complete DMEM, and incubated at 37°C in a humidified chamber with an atmosphere of 5% CO_2_. Cells were cultured with either medium alone, 2.5 µg/ml of individual recombinant MAP proteins, or 5 µg/ml MAP whole cell lysate. At 24 h post-stimulation, cells in suspension were collected, centrifuged for 7 min at 300 x g, and the supernatant discarded. One ml of TRIzol™ Reagent (Thermo Fisher Scientific, Inc.) was added to each well to detach and lyse adherent cells, and subsequently used to re-suspend the cell pellet from the corresponding well. Samples were incubated at room temperature for 10-15 min before storing at -80°C.

### Preparation of MAP Whole Cell Lysate

Whole cell lysate was prepared from a log-phase culture of MAP strain gc86 cultured in Difco Middlebrook 7H9 Broth supplemented with 10% Middlebrook OADC enrichment (BD and Company, USA) and 2 mg/L ferric mycobactin J (Allied Monitor Inc., USA). MAP cells were pelleted by centrifugation for 5 min at 12,000 x g, suspended in ice-cold lysis buffer [PBS, 5% glycerol, 5 mM EDTA, 1mM PMSF] and homogenized 5 x 25 s with 0.1 mm Zirconia/Silica beads using a Mini-Beadbeater-16 (Bio Spec Products Inc.). Protein concentration of the clarified supernatant was quantified using a Pierce BCA Protein Assay Kit (Thermo Fisher Scientific, Inc.) and aliquots stored at -20°C.

### RNA Extraction From PBMCs and Mucosal Leukocytes

Cells lysed and suspended in TRIzol™ Reagent were extracted once with chloroform (0.2 ml/ml TRIzol™ Reagent) and RNA isolated from the aqueous phase using the RNeasy Mini Kit (Qiagen, Inc.) as per the manufacturer’s instructions. RNA integrity, quality and quantity were assessed using an Aglient 2100 BioAnalyzer and Nanodrop™ Spectrophotometer (Thermo Fisher Scientific, Inc.). Samples were stored at −80°C.

### Reverse Transcription and Real-Time qRT-PCR

Genomic DNA removal and cDNA synthesis was completed using the QuantiTect Reverse Transcription Kit (Qiagen, Inc.), as per the manufacturer’s instructions. cDNA samples were diluted with RNase-, DNase-free water to a concentration of 4 ng/µl, and stored at −20°C. Real-time qPCR reactions were performed in duplicate with each reaction consisting of PerfeCTa SYBR Green SuperMix™ (Quanta Biosciences, Inc.), 300 nM of gene-specific primers (**Supplementary Table 1**) and 20 ng of cDNA in a final volume of 15 µl. The thermal cycling program was 2 min at 95°C for initial denaturation, followed by 36 cycles of 95°C for 15 s, 60°C for 30 s and 72°C for 30 s, using a Bio-Rad CFX Connect Real-Time PCR Detection System™ (Bio-Rad Laboratories, Inc.). Quantitative threshold cycle (Cq) for each reaction was determined by the CFX Maestro™ Software (Bio-Rad Laboratories, Inc.) and average Cq calculated using arithmetic average of the technical replicates. Average Cq of each sample was normalized to the constitutively expressed gene *YWHAZ* (40). Relative expression was calculated using the equation 2^-ΔΔCq^ as previously described (41).

### Flow Cytometry

The protocol for cell labelling and antibodies for targeting bovine immune cells were previously described (42). Briefly, cryopreserved PBMCs and PP cells were re-suspended to a concentration of 2 x 10^7^ cells/ml in cell-labeling buffer (PBS containing 0.2% w/v gelatin and 0.03% v/v sodium azide), and a 50-µl aliquot added to each well of a U-bottom 96-well plate (Corning, Inc.). An equal volume of primary monoclonal antibody (**Supplementary Table 2**) diluted in cell-labeling buffer was added to each well, incubated on ice for 20 min, and washed three times with cell-labeling buffer. Fluorochrome-conjugated anti-Ig isotype-specific antibodies (**Supplementary Table 2**) were added to each well, incubated on ice in the dark for 20 min, and washed three times in cell-labeling buffer. Cells were fixed using 2% formaldehyde in PBS, and stored at 4°C in the dark. Samples were analyzed with a FacsCalibur (Becton-Dickinson; NJ) using CellQuestPro acquisition and analysis software. A minimum of 10,000 events were captured for each sample and data was collected in list-mode. Isotype controls (**Supplementary Table 2**) and unstained cells were used to monitor the specificity of the cell staining protocol and reagents, and used to set gates for quantifying specific cell populations.

### Serum IgG Antibody ELISA

Serum was prepared from whole blood collected in SST Vacutainers (Becton Dickinson and Company; USA) and aliquots stored at −20°C. Serum samples were collected prior to primary vaccination (Day 0), booster immunization (Day 28), MAP challenge (Day 56), and euthanization (Day 84, 28 days post-infection). The analyst was blinded to treatment groups while performing ELISAs and calculating antibody titers. Immulon™ 2 HB flat bottom 96-well microtiter plates (Thermo Fisher Scientific, Inc.) were coated by adding 100 µl/well of individual recombinant MAP proteins (1 µg/ml) diluted in bicarbonate-carbonate buffer, pH 9.5 and incubated overnight at 4°C. After washing with water, wells were blocked with diluent (Tris-buffered saline [TBS] supplemented with 1% fish gelatin) for 45 min at room temperature (RT). Four-fold serial dilutions of serum (starting at 1:40, in diluent) were added to duplicate wells and incubated at RT for 2 h. For detection of IgG antibodies, alkaline-phosphatase conjugated, goat anti-bovine IgG (1:10,000 in diluent) was added to each well and incubated for 1 h at RT. Each well was reacted with 1 mg/ml p-nitrophenyl phosphate (PNPP) diluted in PNPP buffer (MilliporeSigma Co.) for 2 h at RT. Colorimetric reactions were stopped by adding 70 mM EDTA and absorbance measured at 405 and 490 nm (reference wavelength) using a SpectraMax Plus 384™ Reader (Molecular Devices; USA). Antibody titers were determined using the reciprocal of the highest dilution that resulted in an absorbance value greater than the mean + 2 standard deviations (SD) of the absorbance value from serum samples obtained from Day 0 calves. Calves were also tested for MAP-specific antibodies using the IDEXX Paratuberculosis Verification Ab Test (IDEXX Laboratories, Inc.) as per the manufacturer’s instructions, and completed by a blinded analyst (Prairie Diagnostic Services, SK, Canada). Serum from calves was tested prior to primary vaccination (Day 0), 2 months post-vaccination (i.e., Day 56 - immediately prior to MAP challenge), and at 28 days post-infection. Test results are expressed as an S/P ratio (Sample-to-Positive ratio), and an S/P ≥ 0.55 was interpreted as test-positive.

### Data and Statistical Analysis

GraphPad Prism 8.1 (GraphPad Software, Inc., USA) was used for all data visualization and statistical analyses. Assumptions of normal data distribution were confirmed for all datasets using the Shapiro-Wilk normality test. Leukocyte subpopulation frequencies, cytokine transcript abundance in *ex vivo* re-stimulated cells (PBMCs and PP cells), and serum IgG titers in non-vaccinates and vaccinates were compared using an unpaired, two-tailed Student’s t-test. Bacterial CFU enumeration data was normalized using log transformation, and normality of the data confirmed. All subsequent analyses were applied to the transformed (normally distributed) data. Differences in CFU counts between Silirum vaccinates (n=4) and non-vaccinates (n=5) was determined using an unpaired, two-tailed Student’s *t* test. Two calves in the Silirum vaccine group, and one calf in the non-vaccinate group were removed from the study, prior to MAP challenge/abdominal surgery, due to unrelated health reasons. A one-way ANOVA was applied to compare log transformed CFU counts among non-vaccinates (Group A, n=6) and the three groups vaccinated with recombinant MAP proteins (Group B, n=5; Group C, n=4; Group D, n=6). Multiple comparisons were not performed. One calf in Group B and two calves in Group C were removed from the study, prior to MAP challenge/abdominal surgery, due to unrelated health reasons. *p* values ≤ 0.05 were considered statistically significant.

## Results

### Clinical Observation of Calves Following Surgical Isolation of Intestinal Segments

Daily monitoring of calves post-surgery and MAP challenge revealed no significant changes in body temperature, feed intake, fecal consistency or clinical signs of abdominal pain or discomfort. A total of six calves in both vaccine studies were lost prior to MAP challenge/surgical procedure. All calves successfully recovered from surgery/MAP challenge and were not lost due to surgical complications associated with the abdominal surgery.

### Gross Appearance and Histology of Intestinal Tissue

**Figure 2:**
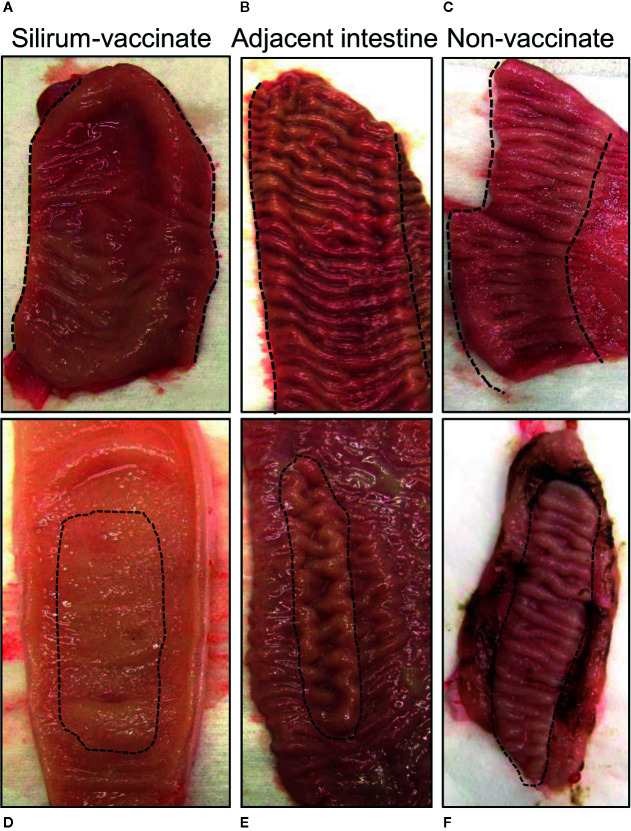
Mucosal surface of the intestinal segments and adjacent intestine. Intestinal segments and adjacent intestine were collected at 28 days post-infection, and the mucosal surface exposed by dissecting along the mesenteric attachment. **(A, B)** Representative images of the mucosal surface of a terminal jejunal segment and adjacent intestine, respectively, from a Silirum vaccinated calf, and a **(C)** terminal jejunum segment collected from an unvaccinated calf. **(D, E)** Representative images of the mucosal surface a mid-jejunal segment and adjacent intestine, respectively, from a Silirum vaccinated calf, and a **(F)** mid-jejunum segment collected from an unvaccinated calf. Hash marks define the margins of the continuous band of PP (CPP) in the terminal jejunum, or the borders of the discrete PP (DPP) in mid-jejunum.

Intestinal segments were removed from the abdomen at 28 days post-infection (84 days post-vaccination) from unvaccinated (n=5) and Silirum vaccinated (n=4) calves. All intestinal segments had visible mesenteric attachments and the serosal surface of each segment was intact. Examination of mid- and terminal-jejunum segments from unvaccinated calves revealed no gross abnormalities or discoloration of the serosal surface compared to adjacent intestine. In contrast, the serosal surface of both mid- and terminal- jejunum segments from Silirum vaccinates showed localized redness, omental adhesions and increased distension; adjoining intestine appeared normal with no visible abnormalities. After opening each segment along the mesenteric attachment, marked differences in the consistency of the luminal contents were apparent when comparing Silirum vaccinates and non-vaccinates. In intestinal segments from non-vaccinates, the luminal contents were a greenish-brown, viscous mass, consistent with our previous observations for MAP infected segments (27–29). By contrast, in Silirum vaccinates the luminal contents in infected segments were less viscous particularly in the mid-jejunal segments, but still greenish-brown in color. Marked differences in the mucosal surface were visible between intestinal segments from Silirum vaccinates when compared to non-vaccinates and to the adjacent intact intestine (**Figure 2**). The mucosal surface in the intestine adjacent to segments in Silirum vaccinates (**Figures 2B, E**) and intestinal segments from non-vaccinates (**Figures 2C, F**) were indistinguishable; both were a uniform pale pink color with no visible signs of mucosal erosion within either the intestinal mucosa or PP, consistent with our previous observations (27–29). In Silirum vaccinates, there was a visible reduction in the prominence of CPP relative to the adjacent mucosal surface (**Figure 2A**) and increased redness of the mucosa with petechiation. Mid-jejunal segments collected from Silirum vaccinates displayed a reduced prominence of DPP (**Figure 2D**) when compared to the surrounding intestinal mucosa (compare **Figures 2D, E**). Further, petechiation was more pronounced in the mid-jejunal segments, specifically in the intestinal mucosa surrounding each DPP.

**Figure 3:**
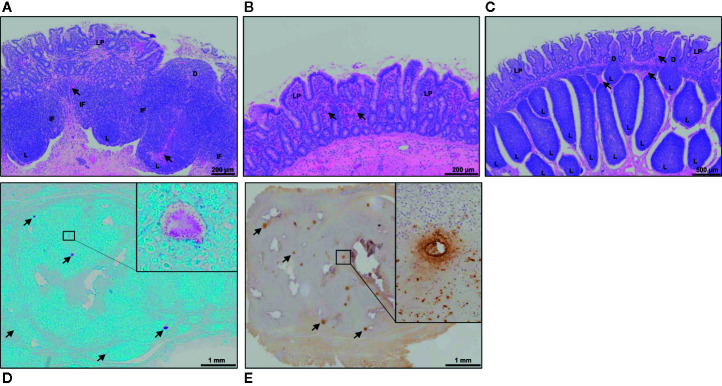
Histology of intestinal tissue and subcutaneous vaccine injection site from Silirum vaccinated calves. **(A)** H&E staining of a discrete PP (DPP) and the **(B)** surrounding mucosa within a mid-jejunal segment, and a **(C)** continuous band of PP (CPP) within a terminal jejunum segment from a Silirum-vaccinated calf at 28 days post-MAP infection. Extravasated red blood cells (arrows) are visible in both mid- and terminal-jejunal segments, specifically within the lamina propria, submucosal lymphoid follicles and interfollicular regions**. (D)** Fite’s acid-fast stain of a tissue section from the encapsulated subcutaneous mass collected at the Silirum vaccine injection at 84 days post-vaccination (28 days post-infection). Multiple foci of red staining (arrows) are dispersed throughout the encapsulated mass. Inset is a digital magnification of a representative foci showing acid-fast bodies and amorphous material present as an aggregate and dispersed among surrounding cells. **(E)** Immunohistochemical stain with polyclonal *Mycobacterium* *avium* ssp. *paratuberculosis* (MAP) antisera of a tissue section from the encapsulated subcutaneous mass collected at the Silirum vaccine injection site. Multiple foci of brown staining (arrows) are dispersed throughout the mass. Inset is a digital magnification of a single foci showing the diffuse brown staining in a localized acellular area and positive staining within surrounding cells. D, dome; IF, interfollicular region; L, lymphoid follicle; LP, lamina propria.

Hematoxylin and eosin (H&E) staining of tissue sections from mid- (**Figures 3A, B**) and terminal-jejunal segments (**Figure 3C**) revealed an intact epithelial barrier, leukocytes throughout the lamina propria within each villus, and follicle-associated epithelium and dome regions above submucosal lymphoid follicles. In mid-jejunal segments, characteristic DPP were present with submucosal lymphoid follicles interspersed among large interfollicular regions populated with lymphocytes (**Figure 3A**). Characteristic CPP were present in the terminal jejunal segments with abundant, closely apposed submucosal follicles with few visible interfollicular regions (**Figure 3C**). Extravasated red blood cells were visible in both mid- and terminal-jejunal segments from Silirum vaccinates, specifically within the lamina propria, submucosal lymphoid follicles and interfollicular regions (**Figures 3A, C**). This was most pronounced in the intestinal mucosa of mid-jejunal segments (**Figure 2B**), consistent with the observed mucosal petechiae. Histological examination of mid- and terminal-jejunal segments from Silirum vaccinates confirmed PPs and intestinal mucosa retained the mucosal architecture and compartmentalization observed in adjacent intestine. Taken together, gross examination and histological analyses of intestinal segments revealed parenteral vaccination with a MAP bacterin results in a localized tissue reaction restricted to the site of enteric MAP infection at 28 days post-infection.

### Gross Appearance and Histology of the Vaccine Injection Site

Subcutaneous injection of the Silirum vaccine induced a local swelling, approximately 7-10 cm in diameter, that was observed in all calves (n=6) but the swelling regressed by 4-8 weeks post-vaccination. This local reaction was consistent with that reported in the product monograph. At 84 days post-vaccination (28 days post-infection) the vaccine injection site contained an encapsulated subcutaneous mass, approximately 2-3 cm in diameter. Upon cut section, the mass consisted of solid tissue with multiple pus-filled foci. Histology of the encapsulated mass revealed numerous foci that stained for both acid-fast bacilli (**Figure 3D**) and MAP protein (**Figure 3E**). This staining provided evidence that the MAP bacterin persisted at the injection site for three months. Furthermore, acid-fast bacteria were evident within and among cells throughout the injection site reaction.

### Bacterial Burden in Intestinal Tissue and Luminal Contents Following Silirum Vaccination and Enteric MAP Challenge

**Figure 4:**
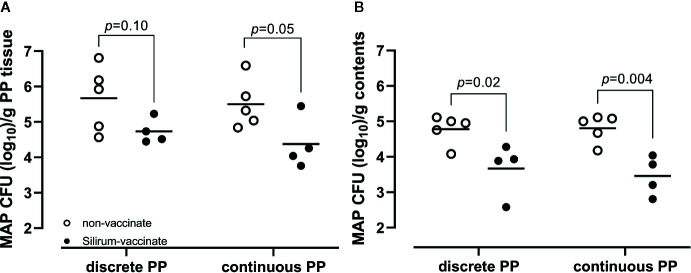
*Mycobacterium avium ssp. paratuberculosis* (MAP) bacterial burden in intestinal tissue and luminal contents. **(A)** MAP recovery from discrete and continuous PP tissue from non-vaccinates (n=5) and Silirum vaccinates (n=4) at 28 days post-infection. **(B)** MAP recovery from the luminal contents of mid-jejunal segments containing a discrete PP and terminal jejunal segments containing a continuous PP from non-vaccinates (n=5) and Silirum vaccinates (n=4) at 28 days post-infection. Data presented are CFU counts from individual animals and the horizontal bar represents the mean value for each group. *p* values determined using a Student’s t-test.

We previously demonstrated that targeted delivery of a defined dose of MAP strain gc86 to intestinal segments, containing either a CPP or DPP, results in consistent and reproducible infection in young calves (29). In the current study, we recovered a consistent level of viable MAP from PP tissue (**Figure 4A**; 5.67 ± 0.92 in DPP and 5.50 ± 0.69 in CPP; mean log10 CFU/g ± 1SD) and luminal contents (**Figure 4B**; 4.78 ± 0.41 in mid-jejunum and 4.81 ± 0.39 in terminal jejunum; mean log10 CFU/g ± 1SD) of unvaccinated calves. This confirmed that persistent infection was established in all animals. Silirum vaccine claims to reduce intestinal bacterial burden. This commercial bacterin was used to determine if a parenteral vaccine could significantly alter MAP burden in our intestinal segment model. There was a numerical decrease (*p*=0.10) in MAP recovered from DPP tissue (**Figure 4A**) and a significant (*p*=0.02) decrease in MAP recovered from the luminal contents of mid-jejunal segments (**Figure 4B**) when comparing Silirum vaccinated versus non-vaccinated calves (n=5). Further, MAP bacterial load was significantly lower in both CPP tissue (*p*=0.05; **Figure 4A**) and the luminal contents of terminal jejunum segments (*p*=0.004; **Figure 4B**) when Silirum vaccinated calves were compared to non-vaccinates (n=5). These data confirm significant changes in MAP burden could be detected in both PP tissue and MAP shed into luminal contents within 28 days post-infection, and the magnitude of these changes was sufficient to identify significant differences between vaccinated and unvaccinated animals.

### Mucosal Leukocyte Populations Following Silirum Vaccination and Enteric MAP Infection

**Figure 5:**
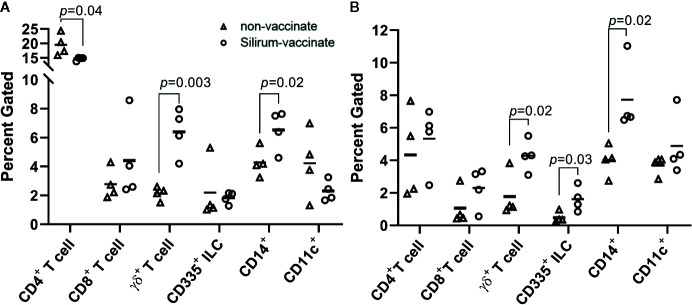
Silirum vaccination alters the frequency of mucosal leukocyte populations following enteric *Mycobacterium avium ssp. paratuberculosis* (MAP) infection. Frequency of T cells (CD4^+^, CD8^+^, γδ^+^), ILCs (CD335^+^), and myeloid cells (CD14^+^, CD11c^+^) in PP cells isolated from **(A)** discrete PP (DPP) and **(B)** continuous band of PP (CPP) of Silirum vaccinates (n=4) and non-vaccinates (n=4) at 28 days post-infection. Each data point represents the percent gated (frequency of cells in 10,000 events) for one calf. Horizontal line represents the mean value for each group; *p* values were determined using a Student’s t test.

The reduction of MAP bacterial burden in intestinal tissue and luminal contents at 28 days post-infection suggested that parenteral vaccination with Silirum significantly altered mucosal immune responses to enteric MAP infection. The reduction in MAP burden was statistically significant in CPP, but not DPP, further suggesting parenteral vaccination may not induce the same effector responses at both intestinal sites. This differential response to vaccination in CPP and DPP provided an opportunity to compare host responses to determine if surrogate markers of immune protection could be identified. Immune cells were isolated from peripheral blood (PBMCs) and the PP tissue (PP cells) within each intestinal segment at 28 days post-infection to analyze the frequency of T cells (CD4^+^, CD8^+^, γδ^+^), innate lymphoid cells (ILCs; CD335^+^) and myeloid cells (CD14^+^ and CD11c^+^). There were no significant differences (*p* > 0.05) in the abundance of T cells, ILCs, or myeloid cells when comparing PBMCs isolated from Silirum vaccinates (n=4) and non-vaccinates (n=5) (**Supplementary Figure 3**). In contrast, Silirum vaccination resulted in distinct changes in mucosal leukocyte populations isolated from the DPP and CPP (**Figure 5**). CD4^+^ T cells were less abundant (*p*=0.04) amongst DPP cells isolated from Silirum vaccinated versus unvaccinated calves (**Figure 5A**) and CD335^+^ ILCs were more abundant (*p*=0.03) in CPP from Silirum vaccinated versus unvaccinated calves (**Figure 5B**). In both the DPP and CPP of Silirum vaccinates, γδ^+^ T cells (*p*=0.003 and *p*=0.04, respectively) and CD14^+^ myeloid cells (p=0.02 and p=0.02, respectively) were more abundant when compared to non-vaccinates (**Figures 5A, B**). Thus, parenteral vaccination with a MAP bacterin significantly altered mucosal leukocyte populations present during MAP infection in both CPP and DPP.

### Cytokine Responses in Peripheral Blood and Mucosal Leukocytes

**Figure 6:**
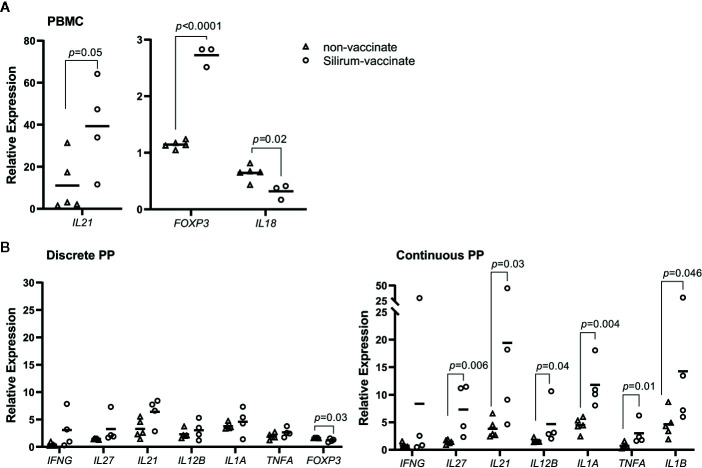
Silirum vaccine-induced cytokine responses in peripheral blood and mucosal leukocytes. **(A)** Peripheral blood mononuclear cells (PBMCs) (5 x 10^6^ cells) and **(B)** PP cells (2 x 10^6^ cells) were isolated at 28 days post-infection from Silirum-vaccinated calves (n=3-4) and non-vaccinates (n=5) and re-stimulated *ex vivo* with *Mycobacterium avium ssp. paratuberculosis* (MAP) whole cell lysate (5 µg/ml) for 24 h. Transcript abundances for 17 cytokine genes (**Supplementary Table 1**) were quantified in resting and re-stimulated cells, and relative expression calculated using 2^-ΔΔCq^. Each data point represents the relative expression for an individual calf; horizontal line represents the mean value for each group. *p* values were determined using a Student’s t test.

Cytokine responses following vaccination and MAP challenge were analyzed to determine if differential responses were associated with the significant reduction in MAP burden in CPP versus less effective vaccine-induced control of enteric MAP infection in DPP. Further, we wanted to determine if vaccine-induced mucosal effector responses in CPP and DPP were similar to responses detected in blood. PBMCs and PP cells, isolated at 28 days post-infection, were re-stimulated *ex vivo* with MAP whole cell-lysate and using real-time qRT-PCR, transcript abundances were quantified for 17 cytokines (**Supplementary Table 1**), representing key cytokines associated with Th1, Th2 and Th17 responses. Responses were compared between Silirum vaccinates and non-vaccinates at 28 days post-infection. In PBMCs, whole cell lysates revealed the induction of greater antigen-specific *IL21* (*p*=0.05; 40-fold increase) and *FOXP3* (*p* < 0.0001; 2.5-fold increase) recall responses, and a significant (*p*=0.02) reduction in *IL18* recall responses in Silirum vaccinates (n=3–4) when compared to non-vaccinates (n=5; **Figure 6A**). In DPP cells isolated from Silirum vaccinates, MAP whole-cell lysate stimulation induced a significant (*p*=0.03) decrease in *FOXP3* when compared to non-vaccinates (n=5; **Figure 6B**) but no significant differences were observed in the expression of the other 16 cytokines assayed. Stimulation of CPP cells isolated from Silirum vaccinates (n=4) with MAP whole-cell lysates led to significant increases in *IL1A* (*p*=0.004; 11-fold increase), *IL12B* (*p*=0.04; 4-fold increase), *IL21* (*p*=0.03; 19-fold increase), *IL27* (*p*=0.006; 7-fold increase) and *TNFA* (*p*=0.01; 3-fold increase) transcript abundances when compared to non-vaccinates (n=5; **Figure 6B**). Cytokine expression data provided further evidence that parenteral vaccination with a MAP bacterin significantly altered mucosal immune responses following enteric MAP infection. Furthermore, these data revealed that immune responses in peripheral blood leukocytes do not accurately reflect the mucosal immune responses occurring at the site of MAP infection.

Systemic antibody responses in non-vaccinates and vaccinates were evaluated using the IDEXX Paratuberculosis verification Ab test (**Supplementary Figure 2**) to measure serum IgG antibodies against a proprietary mixture of MAP antigens following pre-absorption of serum antibodies against an environmental mycobacterial protein extract. Test results are reported as an S/P ratio with a threshold of 0.55 deemed seropositive. Unvaccinated calves (n=5) were seronegative pre-vaccination (with the exception of one animal), at Day 56 post-vaccination with the sham vaccine, and at 28 days post-infection (S/P ratio 0.12 ± 0.05; mean ± 1SD). Despite enteric MAP infection confirmed in both the DPP and CPP segments of these calves (**Figure 4**) the commercial ELISA failed to identify these animals as infected at this early time-point consistent with previous findings (43). In three of the four Silirum vaccinates seropositive test results were observed at Day 56 post-vaccination (prior to MAP challenge), with all calves (n=4) testing positive at 28 days post-infection (S/P ratio 0.92 ± 0.45; mean ± 1SD). One calf was seropositive prior to MAP challenge (S/P = 0.582) and remained seropositive both post-vaccination (S/P = 0.61) and post-challenge (S/P = 0.660). These data suggest Silirum vaccination in some animals can induce systemic antibody responses that result in seroconversion. However, larger cohorts are warranted to confirm the frequency of seroconversion induced by Silirum-vaccination. Despite two animals (one in each unvaccinated and Silirum vaccinated group) testing seropositive pre-vaccination, the MAP CFU recovery and immune responses in these animals did not present as outliers when compared to the other animals within their respective cohorts. As such, these calves were retained in the study analysis.

### Screening MAP Subunit Vaccine Candidates With the Intestinal Segment Model

The commercial Silirum vaccine provided direct evidence that parenteral vaccination significantly altered the mucosal immune response to an enteric MAP infection in surgically isolated intestinal segments. We next evaluated the intestinal segment model as a tool for rapid screening of MAP subunit vaccine candidates in the natural host. Groups of five recombinant MAP proteins (50 µg each) were co-formulated as a vaccine candidate and three pools of five recombinant proteins were evaluated (**Table 1**). Cohorts of age-matched calves were randomly assigned to one of four groups (**Figure 1**) receiving either adjuvant alone (Group A, n=6) or one of the three recombinant protein pools (Group B, n=5; Group C, n=4; Group D, n=6). A single intestinal segment containing a DPP was surgically isolated in this experiment to optimize the high-throughput capacity of this vaccine screening model. In contrast to the intestinal segments recovered at 28 days post-infection from Silirum vaccinates, no gross or histological abnormalities were observed when collecting intestinal segments from either the vaccinated groups or non-vaccinate group (data not shown). MAP bacterial burden was quantified in the luminal contents recovered from each segment (**Table 1**). No significant difference in MAP burden (*p*=0.22; one-way ANOVA) was observed when comparing vaccine cohorts with the non-vaccinate controls (**Table 1**). To confirm that the recombinant MAP proteins were appropriately formulated to be immunogenic, serum IgG antibody responses were measured prior to primary vaccination, at the time of booster immunization, on the day of MAP challenge, and at 28 days post-infection. Vaccinated calves developed significantly elevated serum IgG antibody responses to each recombinant protein prior to MAP challenge (**Supplementary Figure 4**) and maintained elevated antibody titers post-infection (data not shown) when compared to time-matched samples collected from the non-vaccinate group. Antibody responses confirmed that recombinant MAP proteins were immunogenic and induced isotype-switched B cell responses (**Supplementary Figure 4**).

We further examined systemic antibody responses using the IDEXX Paratuberculosis Verification Ab test (**Supplementary Figure 2**). Immediately prior to vaccination, the majority of calves (n=18) were seronegative; three calves (one from the non-vaccinate group and one from each of Group B & D vaccine groups) were seropositive (S/P ratio of 0.76, 0.71, and 0.74 respectively). Consistent with the unvaccinated calves in the Silirum vaccine study, all the unvaccinated calves in this cohort (n=6) were seronegative both prior to MAP challenge (Day 56; S/P ratio 0.11 ± 0.08; mean ± 1SD) and at 28 days post-infection (S/P ratio 0.09 ± 0.06; mean ± 1SD). The one unvaccinated calf that was seropositive prior to vaccination was seronegative prior to MAP challenge (Day 56) and at 28 days post-infection. All Group B vaccinates (n=5), including the one calf that was seropositive prior to vaccination (S/P ratio 0.71), were seronegative prior to challenge (Day 56) and at 28 days post-infection. All Group C vaccinates (n=4) were seropositive both prior to challenge (Day 56; S/P ratio 1.53 ± 0.21; mean ± 1SD) and at 28 days post-infection (S/P ratio 1.11 ± 0.29; mean ± 1SD). Antibody responses in Group D vaccinates were more variable. Two calves remained seronegative throughout the trial. The one calf seropositive prior to vaccination (S/P ratio 0.74) was also seropositive prior to MAP challenge (Day 56), and reverted to seronegative at 28 days post-infection. Four Group D calves were seropositive prior to challenge (Day 56; S/P ratio 1.36 ± 0.81; mean ± 1SD) but responses appeared to wane at 28 days post-infection (S/P ratio 0.72 ± 0.49; mean ± 1SD). Similar to the findings in the Silirum vaccine study, systemic antibodies measured using this commercial ELISA correspond with parenteral vaccine-induced responses, and not responses induced by enteric MAP infection.

We also examined MAP protein-specific cytokine responses in PBMCs and PP cells to determine whether parenteral vaccine-induced effector cells were recruited and retained at the site of MAP infection in PPs. Expression of *IFNG*, *IL17A*, *IL12B*, and *IL27* was analyzed with PBMCs and PP cells isolated at 28 days post-infection and re-stimulated with individual recombinant MAP proteins. Due to the lack of clearly defined correlates of protection for mycobacterial diseases *IFNG*, *IL17A*, and *IL12B* were selected based on their central importance in mediating cell-mediated immunity; *IL27* is a surrogate marker of protection identified in our previous study (29). Antigen-specific cytokine recall responses within each vaccine cohort (Group B, n=5; Group C, n=4; Group D, n=6) were compared to responses induced by the same proteins when stimulating cells isolated from non-vaccinates (n=6) (**Table 1**). When PBMCs were analyzed, *IL17A* recall responses (*p* < 0.05) were evident for 9 proteins (MAP0367, MAP3836c, MAP1981c, MAP4143, MAP1588c, MAP4142, MAP3968, MAP0144, and MAP4263), and only one protein, MAP4143, induced a significant *IFNG* recall response (*p*=0.044). None of the 15 recombinant MAP proteins induced significant changes in the transcript abundance of *IL12B* or *IL27* when comparing PBMCs isolated from vaccinates versus non-vaccinates. Consistent with PBMC recall responses, MAP4143 also induced a significant *IFNG* response (*p*=0.0005; n=5) in PP cells isolated from vaccinated versus unvaccinated animals (n=6). For the remaining 14 proteins, recombinant protein-induced cytokine responses in PP cells did not match responses observed in PBMCs. For example, in Groups B and C, 7 of the 10 proteins induced increased *IL17A* recall responses in PBMCs but only one of these 7 proteins (MAP1981c) induced a significant *IL17A* recall response in PP cells and transcription was repressed in vaccinates when compared to non-vaccinates. The lack of recombinant protein-induced recall responses in PP cells may suggest a failure of antigen-specific effector cells induced in the periphery to be recruited or retained at the site of MAP infection. For vaccinate Group D, however, there was evidence for all 5 proteins significantly altering either *IL17A, IFNG or IL12B* recall responses in PP cells when comparing the vaccinate to non-vaccinate groups (**Table 1**). These data support the conclusion that antigen-specific effector cells could be recruited to and retained at the site of infection. These findings emphasize that interrogating mucosal immune responses at the site of infection provides a very different understanding of parenteral vaccine-induced immune responses than responses monitored with blood leukocytes.

## Discussion

The need for a bovine MAP infection model is evident in the differences in MAP infection and immunopathology in surrogate animal models, including closely related small ruminants. Oral challenge of goats (22, 23) and lambs (44) with a Type II cattle MAP strain revealed greater MAP tropism for DPP and other major mucosal immune induction sites (i.e., ileocecal valve PP and proximal colon PP) than the CPP. In contrast to goats, the CPP is the predominant site of persistent MAP infection in cattle (29, 33, 45, 46). Pathogen-induced immunopathology is also vastly different in goats and calves challenged with Type II cattle strains. In goats, MAP infection of DPP resulted in excessive tissue inflammation and lesion development in the PPs and surrounding intestinal mucosa (22, 23). This observation is not consistent with our observations of MAP infection in the DPP of calves at 12 months post-infection (29), or naturally MAP-infected cows (25). Furthermore, the failure of caprine DPP to control MAP infection differs from our observations that MAP-infection was controlled in bovine DPP at 12 months post-infection with no development of lesions (29). These marked species differences emphasize the need for a model in the natural host, specifically young calves, to test vaccine candidates and assess protection against bovine paratuberculosis. For ovine or caprine paratuberculosis, a vaccine is needed to enhance protective immune responses in major mucosal immune induction sites, whereas in cattle a vaccine should enhance protective immune responses in CPP. This is a unique challenge since the CPP functions as a primary lymphoid tissue but an acquired immune response is necessary for control of MAP infection.

This is the first report of a MAP vaccine-screening model in cattle that provides a quantitative read-out of enteric MAP infection (**Figure 4**). In the current study, targeted delivery of MAP to intestinal segments resulted in infection in all segments of all calves (**Figure 4**), consistent with results from challenge studies in a much larger number of animals (29). A MAP challenge model in which infection is consistently established in all animals is critical to achieve quantitative read-outs such as MAP burden in intestinal tissues and luminal contents, which are relevant metrics for screening vaccine efficacy. Although the oral route of infection best mimics the natural route of exposure, in an experimental animal model this leads to random uptake and non-uniform distribution of infection across the gastrointestinal tract. In turn, this results in a number of undefined variables, including: (i) The site(s) of infection, (ii) Infectious dose at any given intestinal site and, (iii) Whether a test-negative intestinal site was exposed to MAP but subsequently cleared or controlled infection. Moreover, MAP shedding, which occurs intermittently days, weeks, and months post-infection (33, 34) facilitates further exposure and possible re-infection, compounding variability in infected tissues such as bacterial load in each tissue and the resulting induction of host immune responses. Collectively, these parameters compromise quantitative comparisons of bacterial burden and mucosal immune responses at different intestinal sites within an animal and among animals. This is demonstrated by the large inter-animal variance in the number of tissues colonized and associated bacterial load reported in many MAP infection and vaccine screening experiments in goats, sheep and calves (11, 19–21, 23, 30–32) as well as MAP fecal shedding, antigen-specific IFNG responses and serum IgG antibody titers (23). Consistent levels of infection provides greater power when using relatively small numbers of animals to measure MAP disease protection. The use of small cohorts of animals to evaluate vaccine efficacy is also important given the substantial cost of using large animals for research.

This is the first published study, to our knowledge, to investigate Silirum^®^ vaccination in young calves, the intended target species for this vaccine. Previous studies with the Silirum vaccine in goats (11) and farmed red deer (47) reported significant reductions in MAP fecal shedding in vaccinates. Consistent with these findings, we found that intestinal segments from Silirum vaccinates had significantly less MAP in the luminal contents when compared to non-vaccinates (**Figure 4B**). Only two studies have investigated similar (bacterin-based) parenteral vaccines in cattle. Mycopar^®^ vaccination of dairy cattle herds was shown to reduce MAP fecal shedding (16) while Gudair^®^ vaccination of young calves demonstrated the potential of MAP bacterins to induce host responses that interfere with bovine tuberculosis testing (12). The vast majority of studies on parenteral MAP bacterins (e.g. Mycopar or Gudair) or live-attenuated strains have been completed in sheep and goats. A common finding among these reports is that parenteral vaccination of cattle and small ruminants with MAP bacterins can reduce MAP shedding in feces, but is not effective in preventing initial infection or inducing sterilizing immunity (9, 48). Consistent with this, we observed that Silirum vaccination could not prevent infection, but significantly reduced MAP burden in the CPP, but not DPP, at 28 days post-infection (**Figure 4A**). This observation supports the conclusion that parenteral vaccination with a bacterin induced immune responses that significantly altered the host-MAP interaction at the site of infection in the CPP. This provides direct evidence that a parenteral vaccine can alter the mucosal immune response to an enteric infection.

An analysis of MAP-specific immune responses in peripheral blood leukocytes provided an opportunity to determine whether systemic immune responses reflect immune responses at a mucosal site where MAP infection is being controlled. In the current study, we did not detect any differences between Silirum vaccinates and non-vaccinates when analyzing the frequency of T cells (CD4^+^, CD8^+^, γδ^+^), ILCs (CD335^+^), or myeloid cells (CD14^+^ or CD11c^+^) in PBMCs at 28 days post-infection (**Supplementary Figure 3**). Conversely, significant changes in these cell populations were identified in mucosal leukocytes isolated from the site of infection (**Figure 5**). Furthermore, antigen re-stimulation of PBMCs and mucosal leukocytes demonstrated that peripheral blood responses were not similar to those observed at the site of MAP infection (**Figure 6**, **Table 1**). Few studies have investigated mucosal immune responses following MAP infection, and none have investigated whether systemic immune responses correspond with mucosal responses. Responses in peripheral blood have been difficult to detect early after MAP infection (23, 45, 49, 50), which is expected considering the localized, asymptomatic nature of enteric infection and minimal changes in the host mucosal response reported at 12 h and up to two months post-infection (28, 29, 51–54). In previous vaccine studies, changes in the frequency of immune cells in PBMCs have been observed at later time-points post-infection. In calves vaccinated with a cocktail of recombinant MAP proteins, PBMCs collected at 8 weeks post-oral challenge and re-stimulated *in vitro* showed an increase in the abundance of antigen-specific CD3**^+^**, CD8β**^+^**, γδ**^+^** and CD25**^+^** T cells (19). Similar changes in T cell subsets (CD4**^+^**, CD8**^+^** and γδ**^+^**) were identified in *in vitro* re-stimulated PBMCs collected from calves at 11 weeks post-oral challenge following vaccination with a viral-vectored vaccine (20). In Gudair-vaccinated sheep, vaccine-protected sheep displayed increased antigen-specific proliferation of peripheral blood CD4**^+^** and γδ**^+^** T cells, and B cells at 13-21 weeks post oral MAP challenge when compared to Gudair-vaccinated sheep that did not control infection (55). In contrast to these studies, we did not investigate changes in cell frequency following *ex vivo* antigen re-stimulation, and our time-point of analysis (28 days versus 8 to 21 weeks post-infection) was much earlier; both variables that may have reduced the probability of detecting peripheral blood responses. Our rationale for selecting an earlier endpoint than prior studies was to test the utility of our model to more rapidly screen for vaccine-induced responses at the enteric site of infection and not in peripheral immune compartments.

Despite the ease of sampling and measuring responses in peripheral blood, PBMCs provide a poor proxy of mucosal immune responses, especially when screening vaccine-induced responses relevant to enteric infection. Analysis of cytokine expression responses to individual recombinant MAP proteins revealed vaccine-induced responses measured with PBMCs did not reflect any of the responses detected with leukocytes isolated from the enteric site of infection (**Table 1**). This indicates that either recruitment to or retention of antigen-specific effector cells at the site of infection alters the local effector response to a vaccine antigen. As blood contains cells trafficking from the mucosal site of infection as well as those emerging from the vaccine immune induction site it is impossible to discriminate between contributions made by each immune compartment to vaccine antigen recall responses. Intestinal segments provide a means to directly measure antigen-specific effector cells recruited, retained and activated at the site of MAP infection.

The subunit protein vaccine candidates in this study did not elicit a protective immune response that reduced intestinal MAP burden at 28 days post-infection (**Table 1**). Serum antibody responses (**Supplementary Figure 4**) confirmed all vaccine candidates were immunogenic but we also quantified protein-specific mucosal immune responses at the site of infection to confirm that parenteral immunization induced a mucosal response at the site of MAP infection. *Ex vivo* recall responses with individual recombinant proteins confirmed specific responses were present at the site of infection in vaccinated animals (**Table 1**). Analysis of cytokine responses revealed some vaccine antigens induce an *IFNG* response but for other vaccine antigens a decreased cytokine response was observed when compared to non-vaccinates. Similar to our observations in the Silirum vaccine study, vaccine-induced immune responses in blood (i.e., PBMCs) were not consistent with responses occurring at the site of infection (**Table 1**) demonstrating that vaccine immunogenicity measured with blood leukocytes does not provide an accurate indication of mucosal responses at the site of infection. When evaluating vaccine efficacy, it is critical to determine if vaccine antigens failed because they were not immunogenic or failed to elicit protective immune response following infection. Our model provides a unique opportunity to address these questions in the intended target population. With respect to our candidate vaccines, it is evident the vaccine antigens were immunogenic and antigen-specific leukocytes were recruited to the site of infection. However, vaccine formulation may require further optimization to enhance the magnitude or quality of the effector cells recruited to the site of infection. An additional explanation for the failure of the recombinant protein vaccines is that a longer time-interval between MAP challenge and tissue collection may be required to generate sufficient effector cells to control MAP infection in the intestine. Further studies are required to determine if increasing the post-infection interval results in a progressive reduction in MAP bacterial burden and an increased abundance of antigen-specific effector cells at the site of infection. An increased post-infection interval may also provide insight into the durability of protective immune responses, ensuring vaccination provides sustained immunity. Further studies to optimize the post-infection interval may enhance model sensitivity and validate the capacity of the model to identify vaccine candidates that will provide long-term immune protection.

Another aspect of the study that warrants further consideration is the criteria used to exclude calves that may have been exposed to MAP. The absence of a regional Johne’s disease control program did not allow us to source calves from MAP negative herds. To minimize exposure to MAP, calves were sourced from a local supplier who obtained newborn calves within 24 h of birth, housed calves individually, and fed a commercial milk replacer. In previous vaccine studies, commercial ELISAs were used to pre-screen neonatal calves for maternal MAP-specific antibodies as a proxy for possible MAP exposure from the dam. In the current study, the commercial ELISA failed to detect an antibody response in the unvaccinated groups at 28 days post-infection (**Supplementary Figure 2**). This result is consistent with that observed in a previous oral infection study with young calves (43). These data confirm that pre-screening with a commercial ELISA is not effective to determine enteric MAP infection in young calves since a negative result at this time does not preclude infection. In our study, calves that were seropositive pre-vaccination did not show significant alterations in MAP bacterial burden or host immune responses when compared to other calves in their treatment group. The lack of an apparent effect may reflect the short post-infection interval in our model. This does not preclude the possibility that MAP exposure at birth may have significant effects in longer-term infection studies. Pre-screening based on MAP fecal shedding presents an alternative exclusion criterion; however, controlled infection studies have demonstrated significant limitations in the early detection of fecal shedding in young calves (34). Furthermore, culturing MAP requires several months to visualize colony forming units, which would substantially delay testing of candidate vaccines in neonatal calves – a primary target population for MAP vaccine programs. For our intestinal segment model, future studies might include culturing MAP from an intestinal biopsy collected from the surgery site or including a second uninfected segment to perform paired analyses to better control for potential MAP exposure. In spite of these challenges, our short-term infection model allowed us to identify significant differences in both MAP bacterial burden and mucosal immune responses using conventionally reared calves following Silirum vaccination. These statistically significant differences were apparent despite some variance in the challenge dose when comparing among cohorts (**Supplementary Figure 1**). Collectively, the Silirum vaccine served as an invaluable tool, benchmark, and positive control for vaccine-challenge studies in young calves. The purpose of our model is to provide an economical and relatively rapid platform in the natural host to screen and select candidate vaccines for further testing in larger cohorts, using longer post-infection intervals or field trials.

Differential control of enteric MAP infection in the DPP and CPP following Silirum vaccination presented an opportunity to compare mucosal immune responses at each site and determine if specific immune responses could be identified in the CPP that correlated with immune protection. Silirum vaccination promoted an increased accumulation of γδ T cells in both the DPP and CPP (**Figure 5**). The extent of protection afforded by γδ T cells responses to MAP infection *in vivo* is undetermined. Persistent MAP infection in CPP of young calves promoted a local accumulation of the WC1^+^ subset of γδ T cells, decreased the WC1^neg^ subset in the intestinal epithelium, and increased the total γδ T cells in lamina propria at 7 months post-infection (39). In the present study, we did not investigate specific γδ T cell subsets. Further study is warranted to determine whether vaccination results in differential expansion of specific γδ T cell subsets in the functionally distinct PPs given that the effector responses of γδ T cells to MAP may be affected by the diverse cytokine and immune cell landscapes within each type of PP (42, 56). A rapid and early accumulation of γδ T cells might be one essential component in controlling MAP infection, specifically in the CPP. *In vitro* co-culture experiments showed that bovine γδ T cells can enhance the MAP bactericidal activity of infected monocyte-derived macrophages, and promote the secretion of IFNG and IL17A (57–59).

In addition to γδ T cells, CD14^+^ myeloid cells were also more abundant in both DPP and CPP of Silirum vaccinates following enteric MAP infection (**Figure 5B**). Phenotypically and functionally diverse subsets of myeloid cells have been described in human and mouse PPs (60) which are functionally similar to the ruminant DPP. However, there are no published studies describing the myeloid populations within the submucosal lymphoid follicles and interfollicular regions of ruminant DPP and CPP. Based on the functional differences between the bovine DPPs (major mucosal immune induction sites) and CPP (primary lymphoid tissue and site of B cell lymphopoiesis) it is plausible that distinct myeloid cell subsets and populations may reside in each tissue. Distinct myeloid and dendritic cell subsets are known to populate the intestinal mucosa of the ileum and jejunum of young calves (42, 61). As this was a single marker analysis of myeloid cells, it is unknown whether Silirum vaccination led to phenotypically-altered or functionally-similar myeloid cell subset(s) in both DPP and CPP. Further analysis is needed to define the phenotype of the changed myeloid cell populations to determine if they differ in their capacity to harbor, or control, intracellular MAP infection.

In Silirum vaccinates, CD4^+^ T cells were significantly less abundant in the DPP (**Figure 5A**) when compared to non-vaccinates, but no changes in CD4^+^ T cells were detected in the CPP. T helper cells are essential for orchestrating cell-mediated immune responses against intracellular pathogens, including mycobacteria. The reduction of CD4^+^ T cells in the DPP may have contributed to a less effective control of MAP infection in DPP as compared to the CPP. Furthermore, it is intriguing that parenteral vaccination resulted in the reduction of T cells at a major mucosal immune induction site. Whether this is a transient response or a permanent impact on the mucosal T cell population requires further investigation as this adverse effect could compromise mucosal immunity to other invading pathogens.

CD335^+^ ILCs were more abundant in the CPP of Silirum vaccinates following enteric MAP infection when compared to non-vaccinates. ILCs have not received much attention in the context of mucosal MAP infection. A recent study identified an increased frequency of CD335^+^ leukocytes in antigen re-stimulated PBMCs isolated from lambs, goats and calves at 12 months post-MAP infection (26). The importance of ILCs, such as Natural Killer (NK) cells, in promoting protective immune responses against *Mycobacterium tuberculosis* (MTB) infection has been demonstrated in mouse models and human cell culture systems (62, 63). In *Mycobacterium bovis* BCG-vaccinated infants, adults with latent TB (64), and in mouse models of MTB infection (65), vaccine-induced NK cell responses correlated with protection. Additional studies have demonstrated that NK cells mediate protective responses to MTB through their interactions with CD8^+^ T cells (66) and γδ T cells (67), and by directly engaging with infected macrophages (68, 69). NK cell effector functions are associated with IL15, IL18, IL27 (70, 71), and, specifically in the context of MTB infection, with IL21 (65), IL12 (72), TNFA (72, 73) and IL22 (70). Vaccine-induced cytokine responses in the CPP of Silirum vaccinates reflected similar cytokine responses (*IL12*, *IL21*, *IL27* and *TNFA*) that have previously been associated with NK cell activity. Taken together, these data suggest that pathogenic mycobacteria (i.e., MAP and *M*. *bovis*) share common features effective in eliciting ILC responses during vaccination, with clear evidence in MTB infection models that these responses are protective. Further investigation of ILCs, and their effector cytokine responses, in the context of enteric MAP infection of ruminants is warranted.

Vaccine-induced protection in Silirum-vaccinated calves provided an opportunity to further investigate antigen-specific cytokine responses associated with control of MAP infection. In the current study, vaccination of calves with Silirum resulted in significant control of MAP infection in the CPP, but not DPP (**Figure 4**). This is the inverse of our observations with MAP infection of the CPP and DPP at 12 months post-infection albeit in the absence of vaccination (29). In both studies, independent of the intestinal site of infection, control of MAP infection correlated with local antigen-specific *IL27* responses providing evidence this cytokine might provide a correlate of immune protection. In addition to *IL27*, local antigen-specific cytokine responses for *IL1A, IL1B, IL12B*, *IL21* and *TNFA* were detected in the CPP of Silirum vaccinates (**Figure 6**). *IL1A*, *IL1B*, *IL12B* and *TNFA* are key cytokines known to direct protective immune responses against intracellular mycobacteria, including MTB (74). Interestingly, there was no evidence to support the contribution of *IFNG* or *IL17A* when analyzing responses in Silirum vaccinated calves. Other MAP infection and vaccine studies have similarly failed to identify these cytokine responses as correlates of MAP protection (20, 29, 55). Despite the importance of these cytokines in promoting cell-mediated responses, they have not been reliable surrogate markers of immune protection against enteric MAP infection.

To our knowledge, this is the first study showing a possible association between *IL21* and the control of enteric MAP infection (**Figure 6**). In mouse models and human tissue culture systems there is a growing body of evidence showing that IL21 is essential for immune protection against MTB. Following MTB challenge, greater pulmonary bacterial burden and pre-mature death were observed in *IL21* receptor (IL21R) gene knockout mice when compared to WT mice (75, 76). In lung tissue of IL21R deficient MTB-challenged mice, the transcript abundance of *IFNG*, *IL1B*, *IL17*, *IL22*, *IL27*, and *TNFA* was significantly reduced, when compared to that in WT mice (77). Further, IL21R knockout led to impaired CD4^+^ and CD8^+^ T cell accumulation in the lungs, including the reduced frequency of IFNG^+^CD4^+^ and IFNG^+^CD8^+^ T cells (76), and similarly reduced their capacity to produce IFNG and TNFA (75). *In vitro* co-culture experiments revealed that IL21 stimulation of human NK cells, but not direct stimulation of MTB-infected monocytes (77), enhanced the anti-mycobactericidal activity of MTB-infected monocytes when compared to that of unstimulated NK cells (76). Further studies demonstrated that IL21 activated NK cells enhanced the production of IFNG and IL1B, and repressed IL10, from monocytes isolated from latent MTB infected individuals and re-stimulated with MTB antigen (77). Taken together, IL21-dependent NK cell effector responses mediate protective responses against MTB. The similarity in these cytokine profiles to that observed in the CPP of Silirum vaccinated calves in the current study warrants further investigation into the NK cell/IL21 axis in the context of mucosal immune responses to enteric MAP infection.

In the current study, we demonstrated that delivery of a defined dose of MAP to intestinal segments provides a bovine infection model that could be used to evaluate the efficacy of a parenteral vaccine. Quantifying MAP burden in the intestinal tissue and the intestinal lumen provides reproducible measures that reveal significant differences between relatively small cohorts (n = 5) of vaccinated and unvaccinated animals. Using the commercial MAP vaccine Silirum and recombinant MAP proteins as subunit vaccines, we demonstrate proof-of-concept that this model can be implemented for relatively rapid screening of parenteral vaccines that alter host responses to enteric MAP infection. Mucosal immune responses to the enteric MAP infection were altered by parenteral vaccination but there was no correlation between mucosal and systemic immune responses detected in blood. This disparity emphasizes the need for a vaccine screening model in which host responses can be measured at the site of MAP infection. Furthermore, the intestinal segment model enabled us to analyze regional differences in MAP control and immune responses in the CPP and DPP following Silirum vaccination. Differential control of MAP infection in the CPP and DPP also provided a framework for investigating mucosal immune responses and effector cell populations in the natural host that correlate with MAP protection. These analyses indicated that *IL27*, *IL21*, and ILCs might be important for vaccine-induced immunity against enteric MAP infection, mirroring similar protective responses observed in MTB infection models. Further work is needed to address later time-points post-infection to evaluate if these responses provide transient or sustained protection. Bovine intestinal segments provides a means to accelerate vaccine discovery and, as importantly, a method to begin directly investigating vaccine-induced mucosal immunity at the site of MAP infection.

## Data Availability Statement

The original contributions presented in the study are included in the article/**Supplementary Material**. Further inquiries can be directed to the corresponding author.

## Ethics Statement

The animal study was reviewed and approved by University of Saskatchewan Animal Care Committee (Protocol #20160076).

## Author Contributions

AF, HT, AP, RH, and PG designed the study. AF, HT, RH, and PG contributed to data analysis and data interpretation. AF, AL, MT, NR, MB, and NA performed the experiments. AF and PG wrote the manuscript. VG, SN, RH, HT, and LM contributed to manuscript preparation. All authors contributed to the article and approved the submitted version.

## Funding

This work was supported by Genome Canada (LSARP #8309), Genome British Columbia (225RVA), University of Saskatchewan and Government of Saskatchewan (Saskatchewan Ministry of Agriculture—Agriculture Development Fund). PG is funded by a Tier I Canada Research Chair in Neonatal Mucosal Immunology provided by Canada Institutes for Health Research (CIHR). RH holds a Canada Research Chair in Health and Genomics and a UBC Killam Professorship. AL is supported by Simon Fraser University’s New Faculty Start-Up Grant.

## Conflict of Interest

The Silirum^®^ vaccine was kindly provided by CZ Veterinaria. CZ Veterinaria had no influence on the study design, data collection, data analysis, or writing of this work. We have filed the individual MAP proteins for patent protection.

The authors declare that the research was conducted in the absence of any commercial or financial relationships that could be construed as a potential conflict of interest.
