# Supplementary material for: A Bovine Enteric Mycobacterium Infection Model to Analyze Parenteral Vaccine-Induced Mucosal Immunity and Accelerate Vaccine Discovery
Source: Front Immunol. 2020 Nov 23;11:586659. doi: 10.3389/fimmu.2020.586659 (PMC7719698; doi:10.3389/fimmu.2020.586659)
Supplement: Supplementary file 2 [file Table_2.docx]

**Supplementary Table 2. Antibodies used for flow cytometric analysis.**

| **Monoclonal antibodies** | | | | |
| --- | --- | --- | --- | --- |
| ***Antibody Target*** | ***Isotype*** | ***Dilution factor*** | ***Target population*** | ***Supplier, Clone*** |
| CD11c | IgM | 1:100 | Dendritic cells, myeloid cells | Bio-Rad, BAQ153A |
| CD14 | IgG1 | 1:200 | Myeloid cells | WSU, MM61A |
| CD4 | IgG1 | 1:200 | T helper cell | Bio-Rad, CACT138A |
| CD8 | IgG1 | 1:100 | Cytotoxic T cell | WSU, CACT80C |
| γδ | IgG2b | 1:100 | γδ T cell | WSU, GB21A |
| CD335 | IgG1 | 1:100 | Natural killer cells, innate lymphoid cells | Bio-Rad, MCA2365 |
| **Fluorochrome-conjugated antibodies** | | | | |
| ***Target*** |  | ***Dilution factor*** | ***Fluorochrome*** | ***Supplier, Clone*** |
| IgM |  | 1:400 | APC | BD Biosciences, 550676 |
| IgG1 |  | 1:200 | PE | Invitrogen, P21129 |
| IgG2b |  | 1:200 | FITC | Invitrogen, M32401 |
| **Isotype control antibodies** | | | | |
| ***Isotype*** | ***Dilution factor*** | |  | ***Supplier, Clone*** |
| IgM | 1:100 |  |  | Invitrogen, MGM00 |
| IgG1 | 1:100 |  |  | Invitrogen, MG100 |
| IgG2b | 1:100 |  |  | Invitrogen, MG2b00 |
