# Supplementary figures and images for: A Bovine Enteric Mycobacterium Infection Model to Analyze Parenteral Vaccine-Induced Mucosal Immunity and Accelerate Vaccine Discovery
Source: Front Immunol. 2020 Nov 23;11:586659. doi: 10.3389/fimmu.2020.586659 (PMC7719698; doi:10.3389/fimmu.2020.586659)

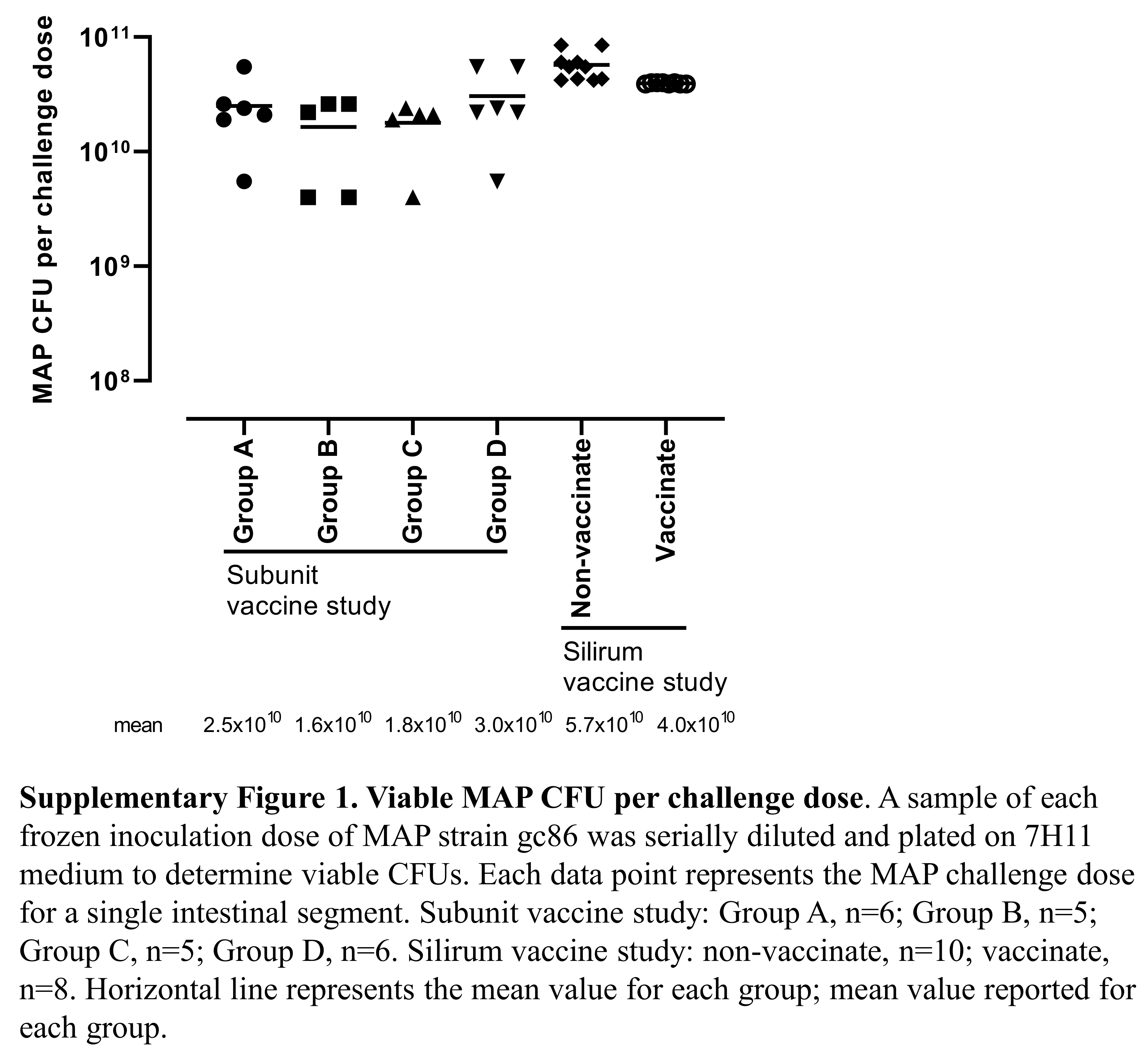

Supplement: Supplementary file 3 [file Image_1.tif]

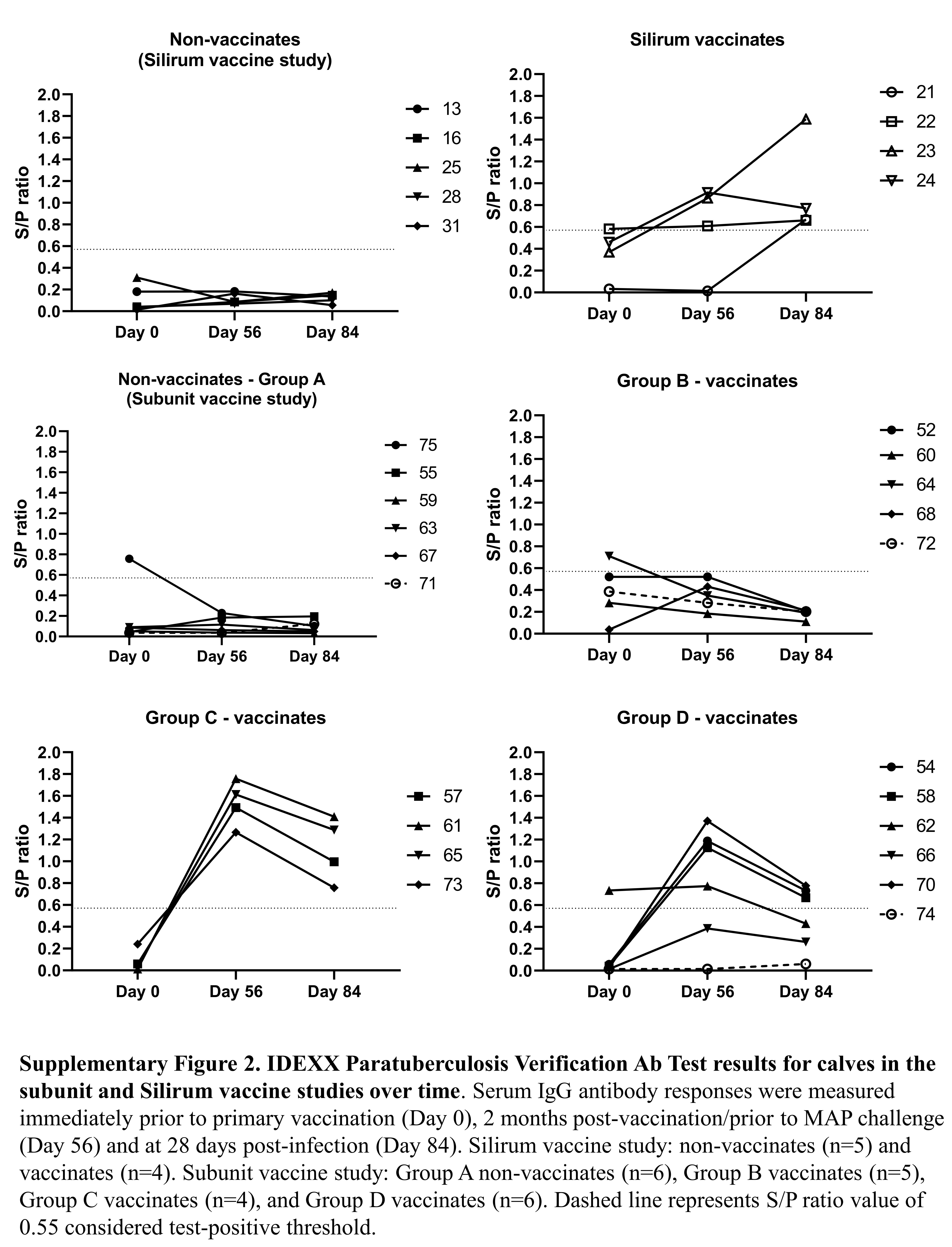

Supplement: Supplementary file 4 [file Image_2.tif]

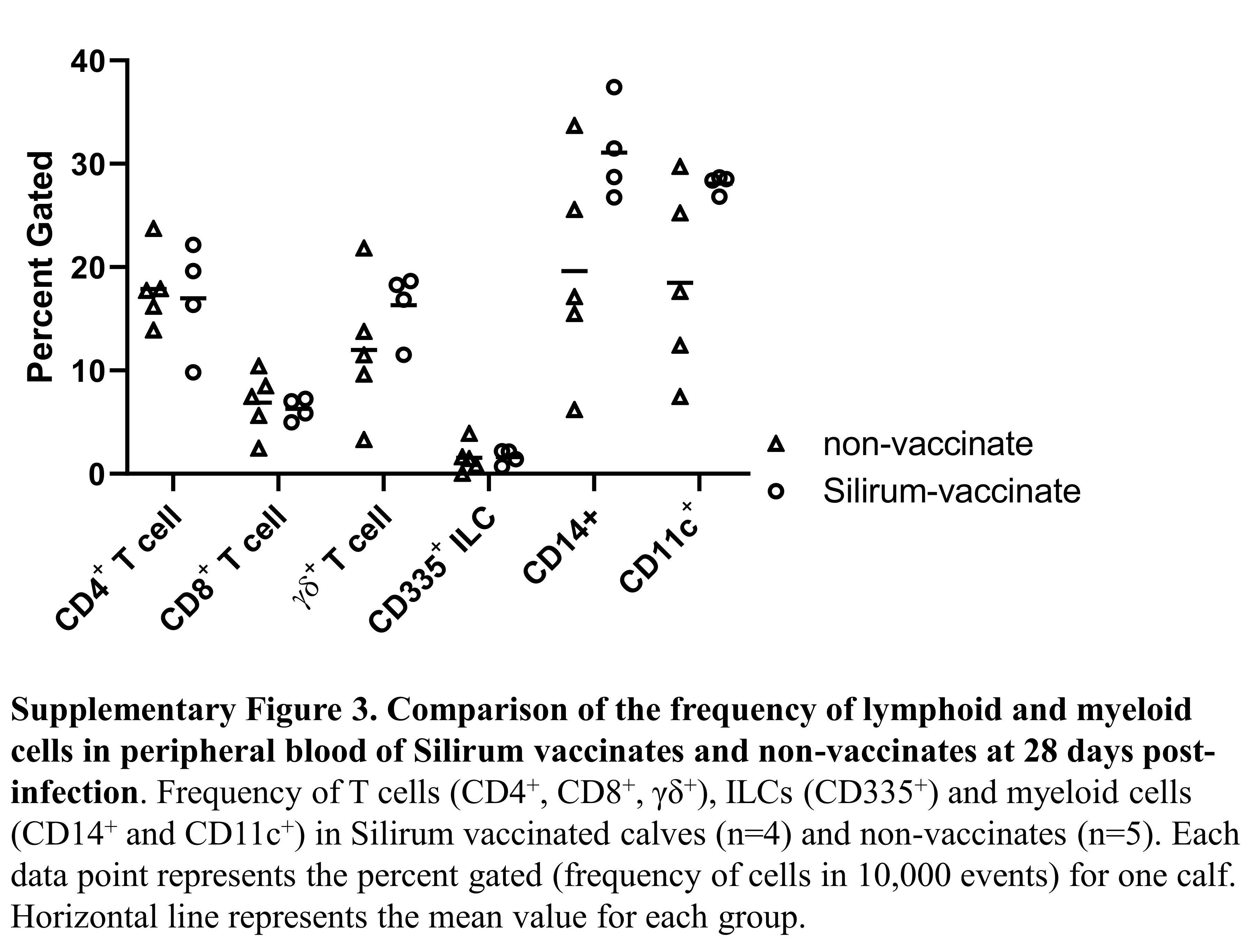

Supplement: Supplementary file 5 [file Image_3.tif]

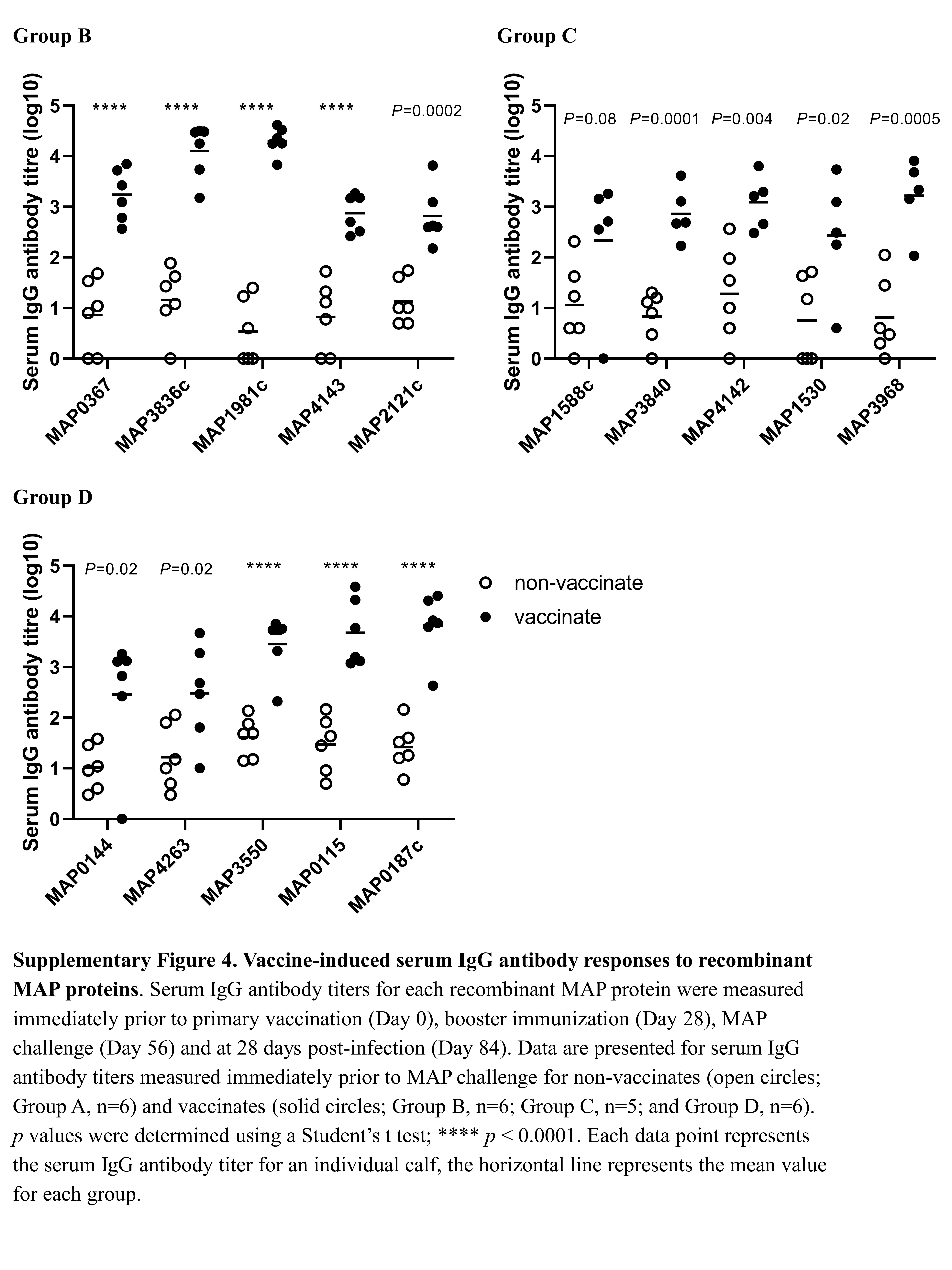

Supplement: Supplementary file 6 [file Image_4.tif]
